# The quality and reliability of Kawasaki disease-related short videos on TikTok and Bilibili: a cross-platform content analysis using GQS, mDISCERN, and JAMA benchmarks

**DOI:** 10.1186/s13052-026-02247-0

**Published:** 2026-03-20

**Authors:** Qiwei Wang, Weiting Jiang, Junwen Wang

**Affiliations:** 1 Department of Gastrointestinal Surgery, Shanghai Baoshan Hospital of Integrated Traditional Chinese and Western Medicine (Baoshan Hospital, Shanghai University of Traditional Chinese Medicine), Shanghai, 201999 China; 2 Department of Gastroenterology, Tongji Institute of Digestive Diseases, Tongji Hospital, School of Medicine, Tongji University, 389 Xincun Road, Putuo District, Shanghai, 200065 China

**Keywords:** Kawasaki disease, Short video, TikTok, Bilibili, Quality and reliability assessment

## Abstract

**Background:**

Kawasaki disease (KD) represents the leading cause of acquired heart disease in children, with timely diagnosis and treatment being crucial to prevent severe coronary complications. The widespread use of short-video platforms such as TikTok and Bilibili has made them increasingly important sources of health information for the public. However, the quality and reliability of KD-related content on these platforms remain largely unassessed. This study aimed to evaluate the educational quality and informational reliability of short videos about KD on TikTok and Bilibili, two of the most popular social media platforms in China.

**Methods:**

We conducted a cross-sectional analysis of the top 100 videos retrieved for KD on each platform. A total of 186 videos were assessed using the Global Quality Scale (GQS), modified DISCERN (mDISCERN), and JAMA benchmark criteria. Uploaders were categorized as professionals or non-professionals. Video characteristics, engagement metrics, and content coverage were analyzed.

**Results:**

Overall quality was suboptimal (median scores: GQS = 2, mDISCERN = 2, JAMA = 2). Videos from professionals scored significantly higher than those from non-professionals (*p* < 0.001). TikTok videos were shorter but had higher engagement and GQS scores than Bilibili videos (*p* < 0.001). Content coverage was skewed: clinical manifestations were discussed in > 80% of videos, while prognosis and prevention were covered in < 25%. Engagement metrics showed negligible correlation with quality scores.

**Conclusions:**

KD-related short videos on popular Chinese platforms exhibit notable quality deficiencies and content gaps. Incomplete or low-quality online information may delay caregiver recognition and timely medical evaluation, potentially increasing the risk of coronary complications in children with KD. Although professional involvement is associated with better quality, overall reliability remains limited. These findings highlight the need for improved content governance and tailored strategies for accurate health communication on social media.

## Background

Kawasaki disease (KD) is an acute, self-limiting systemic vasculitis that represents the leading cause of acquired heart disease in children from developed countries and is being increasingly reported from developing countries too [1, 2]. KD predominantly affects young children, mostly below 5 years of age, with 1.5-times higher risk in boys than girls [3]. Its diagnosis is primarily clinical, based on the presence of characteristic signs including persistent fever, bilateral non-exudative conjunctivitis, changes in the lips and oral cavity, polymorphous rash, cervical lymphadenopathy, and changes in the hands and feet [4, 5]. The most severe complication is the development of coronary artery lesions (CALs), such as aneurysms, which occur in approximately 20–25% of untreated patients and can lead to long-term cardiovascular sequelae, including ischemic heart disease and sudden death [6, 7]. The timely administration of intravenous immunoglobulin (IVIG) within the first 10 days of illness has been shown to significantly reduce the incidence of CALs, underscoring the critical importance of early diagnosis and intervention [5, 8]. Despite extensive research, the etiology of KD remains unknown, with current hypotheses suggesting an abnormal immune response to unidentified infectious or environmental triggers in genetically susceptible individuals [9–12].

The proliferation of digital media has transformed the public’s access to health information. Short-video platforms, notably TikTok and Bilibili in China, have emerged as prominent sources for health education due to their highly visual, engaging, and accessible format [13–15]. These platforms cater to modern information consumption habits, making them potent tools for disseminating knowledge about time-sensitive medical conditions like KD, where parental awareness can directly influence clinical outcomes [16].

However, this rapid dissemination occurs in a largely unregulated space. The absence of standardized peer-review processes and content validation mechanisms on these platforms raises substantial concerns regarding the accuracy, reliability, and comprehensiveness of the health information presented [17]. Prior analyses have documented a high prevalence of misinformation and suboptimal quality in health-related videos across various medical domains, including pediatrics [18–22]. For a condition as nuanced as KD, inaccurate or incomplete information has the potential to mislead caregivers, resulting in delays in seeking appropriate medical care and adversely affecting patient prognosis [23, 24].

Despite the growing reliance on short-video platforms for health information, there remains a notable lack of systematic evaluation of the quality and reliability of KD-related content. To our knowledge, no prior study has specifically assessed the educational value and trustworthiness of KD videos on platforms such as TikTok and Bilibili, despite their widespread use among Chinese caregivers. This gap is particularly concerning given the complexity of KD management and the potential for misinformation to adversely affect patient decision-making and outcomes, especially within the critical 10-day window for IVIG administration.

Therefore, this cross-sectional study aims to perform the first comprehensive assessment of the quality and reliability of KD-related short videos on TikTok and Bilibili. Utilizing validated evaluation tools, the Global Quality Scale (GQS), the modified DISCERN tool (mDISCERN) and the Journal of the American Medical Association (JAMA) benchmark criteria, we seek to: [1] quantify the overall educational quality and informational reliability; [2] compare content characteristics and quality scores between platforms and uploader categories; and [3] identify key informational gaps. The findings from this study will provide a critical evidence base to inform strategies for improving the accuracy of public health communication and guide future content creation and platform governance for pediatric cardiovascular health.

## Methods

### Search strategy and data extraction

A cross-sectional study was conducted to systematically retrieve and evaluate Chinese short videos pertaining to KD. Data were collected from two leading short-video platforms in China: Bilibili (https://www.bilibili.com) and TikTok (https://www.douyin.com) between October 25 to October 28, 2025. The Chinese keyword “川崎病” (KD) was used as the search term on both platforms to simulate a typical patient information-seeking behavior. To ensure the neutrality of the search results and minimize the potential bias introduced by personalized recommendation algorithms, all searches were performed in a logged-out state without using any personal accounts. To reflect typical user exposure under default sorting algorithms, the top 100 videos from each platform were collected, yielding a preliminary dataset of 200 videos.

Videos were excluded from the final analysis for any of the following reasons: (1) Irrelevance: Content was unrelated to KD or only mentioned the term superficially without providing substantive educational or experiential information (2). Promotional Content: Videos identified as advertisements, or those featuring overt self-promotion by healthcare providers or institutions (3). Duplicates: Duplicate videos or re-uploads of the same content from the same uploader (4). Specific Formats: Academic conference recordings or pure online teaching lectures not tailored for a general public audience.

For each included video, the following metadata were extracted: platform (Bilibili/TikTok), URL, uploader identifier, duration (seconds), and engagement metrics (likes, comments, shares, collections).

### Uploader characteristics

The video uploaders were classified into four categories: (1) Specialists (e.g., pediatric cardiologists, rheumatologists); (2) Non-specialists (physicians from other medical fields); (3) Professional institutions; and (4) Individuals (primarily patients). Specialists, non-specialists, and institutions were further collectively termed ‘Professionals’ due to their substantial medical expertise, whereas individuals were designated as ‘Non-professionals’.

### Video quality assessment

Video quality and reliability were evaluated using three validated instruments: the GQS, the JAMA benchmark criteria and the mDISCERN tool [25–27]. The GQS provided a global quality assessment using a 5-point Likert scale (1 = very poor, 5 = very good), based on professionalism, comprehensiveness, clarity, and educational value. The mDISCERN assesses reliability across five criteria (clarity, relevance, traceability, robustness, fairness), each scored dichotomously (1 = yes, 0 = no), yielding a total score of 0–5. The JAMA benchmark criteria assessed four dimensions-attribution, currency, content validity, and disclosure compliance-with composite scores ranging from 0 to 4. Content completeness was evaluated based on coverage of seven key topics: epidemiology, etiology, symptoms, diagnosis, treatment, prevention, and prognosis.

Two independent raters with relevant clinical expertise received standardized training prior to assessment. Inter-rater reliability was quantified using Cohen’s kappa coefficient, with agreement levels defined as follows: κ < 0.4 (poor), 0.4–0.6 (moderate), 0.6–0.8 (substantial), and > 0.8 (excellent). Discrepancies were resolved through consensus or third-party adjudication when necessary.

### Statistical analysis

Data normality was evaluated using the Shapiro-Wilk test. Continuous variables are presented as mean ± standard deviation (SD) for normally distributed data, and as median with interquartile range (IQR) for non-normally distributed data, and categorical variables as frequencies (%). Chi-square tests were utilized to assess differences in platform distributions. Given the non-normal distribution of video length, engagement metrics and quality scores, nonparametric tests were employed: the Mann-Whitney U test for two-group comparisons, and the Kruskal-Wallis test followed by Dunn’s post-hoc analysis for multi-group comparisons. Correlations between video metrics and quality scores were assessed using Spearman’s ρ, with coefficients interpreted as: <0.2 (negligible), 0.2–0.4 (weak), 0.4–0.6 (moderate), 0.6–0.8 (strong), > 0.8 (very strong). All analyses were conducted in IBM SPSS Statistics 23.0, with statistical significance set at *P* < 0.05.

## Results

### Video characteristics

**Fig. 1:**
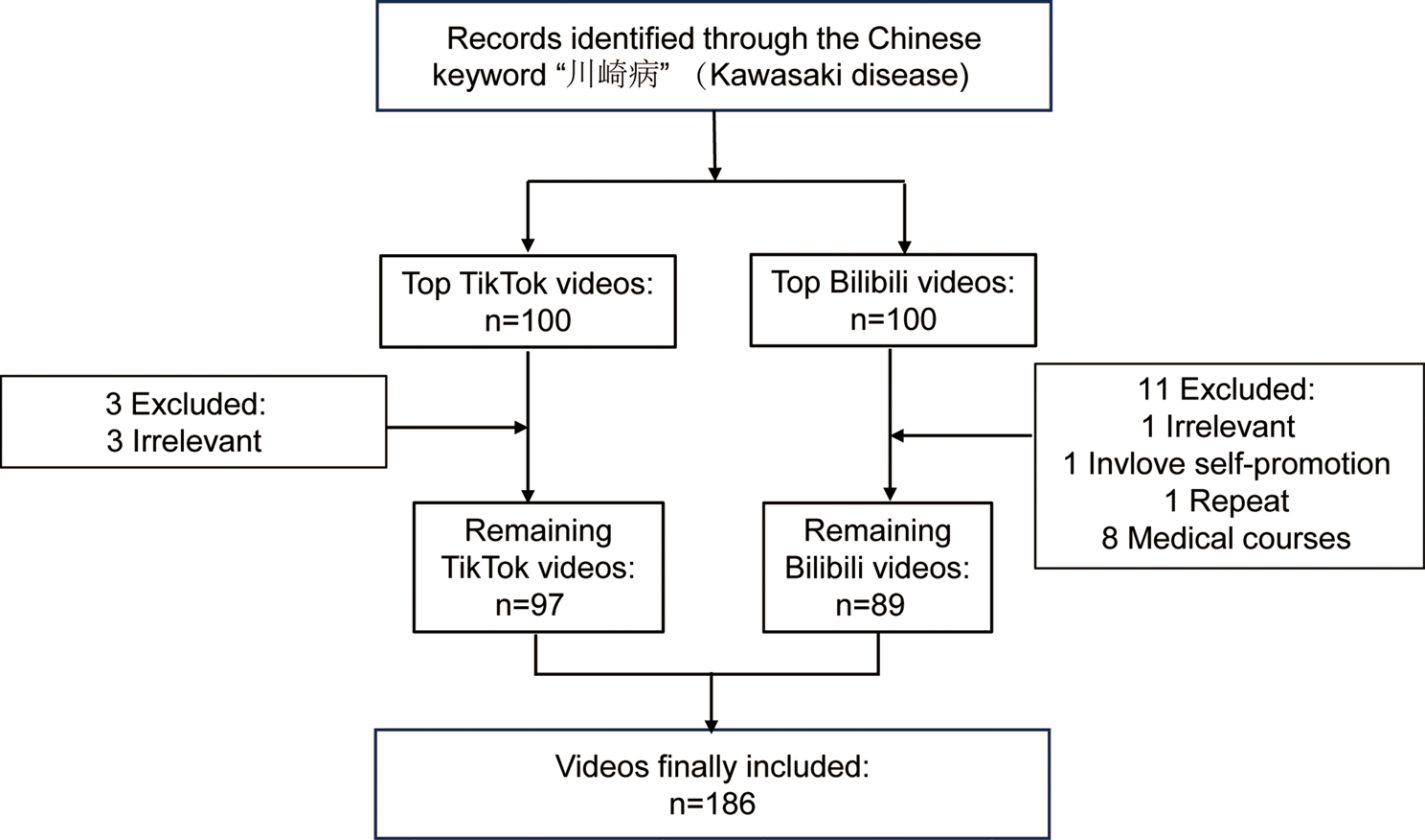
The flow chart of this study

A systematic search on Bilibili and TikTok using “川崎病” identified an initial pool of videos. After applying exclusion criteria, 186 videos (97 from TikTok and 89 from Bilibili) were included in the final analysis (Fig. 1). Video characteristics, including length and engagement metrics, are summarized in Table 1. Overall, videos exhibited modest engagement, with median likes, collections, comments, and shares per video of 261.5 (24.75, 978.75), 75 (10, 429), 35 (3, 213), and 98 (8, 470.75), respectively. The median video duration was 99s (55.5, 213.25). Clinical manifestations were the most frequently covered topic (80.6% of videos), followed by diagnosis (74.7%), treatment (73.1%), etiology (46.8%), and epidemiology (26.3%). Prognosis and prevention were discussed in only 22.6% and 19.9% of videos, respectively. The median GQS, mDISCERN, and JAMA scores were 2 (1, 3), 2 (2, 2), and 2 (1, 2), respectively. Inter-rater consistency was excellent, with Cohen’s kappa values of 0.853 for GQS, 0.867 for mDISCERN, and 0.846 for JAMA scores.

**Table 1:** General characteristics, quality, and reliability of the videos

Variables	Total (*n* = 186)
**General information**	
Video length(s), M (Q1, Q3)	99(55.5,213.25)
Likes, M (Q1, Q3)	261.5(24.75,978.75)
Collections, M (Q1, Q3)	75(10,429)
Comments, M (Q1, Q3)	35(3,213)
Shares, M (Q1, Q3)	98(8,470.75)
**Video content**	
Epidemiology	49(26.3%)
Etiology	87 (46.8%)
Clinical manifestation	150(80.6%)
Diagnosis	139(74.7%)
Treatment	136 (73.1%)
Prevention	37 (19.9%)
Prognosis	42(22.6%)
**Video quality**	
GQS score, M (Q1, Q3)	2(1,3)
mDISCERN score, M (Q1, Q3)	2(2,2)
JAMA score, M (Q1, Q3)	2(1,2)

### Uploader characteristics

**Fig. 2:**
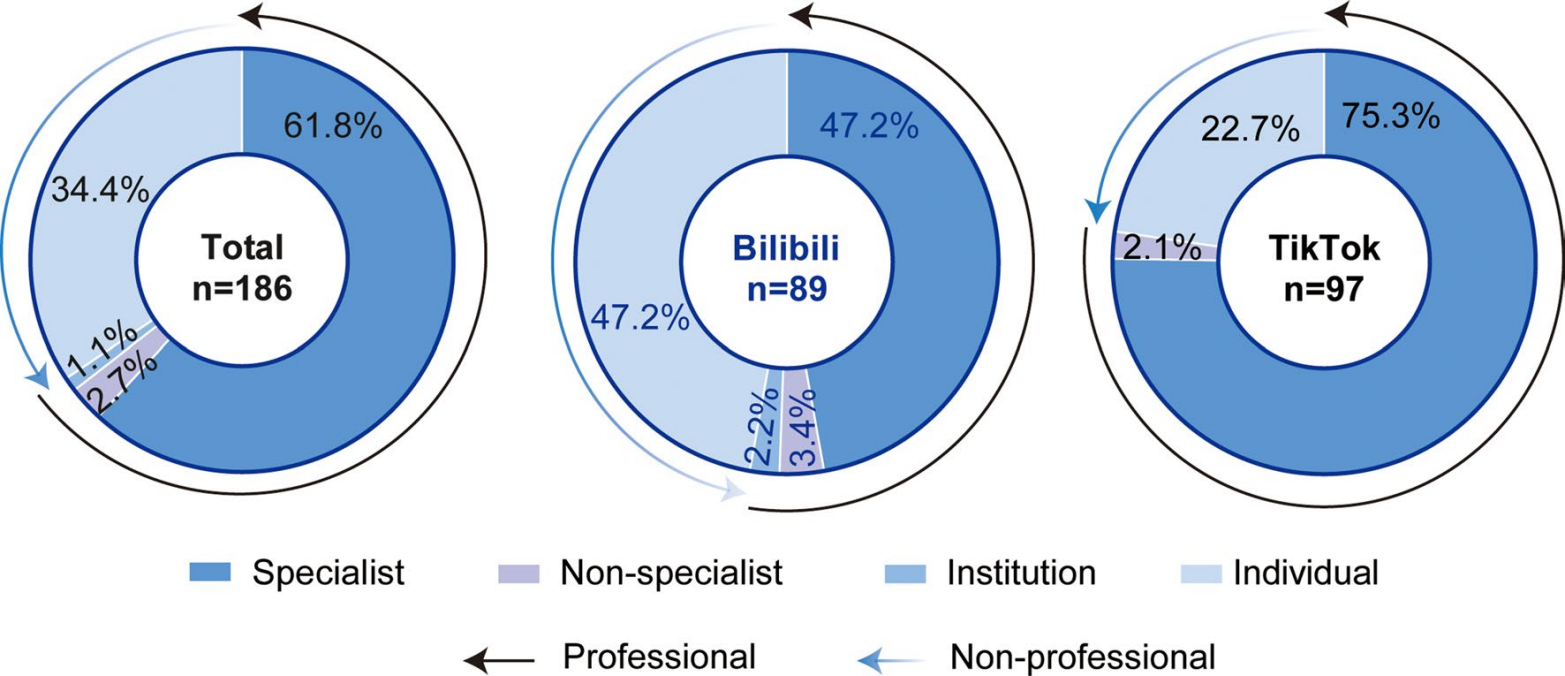
Distribution of video uploaders on Bilibili and TikTok

Uploader distribution is summarized in Fig. 2. Specialists constituted the majority (61.8%), followed by individuals (34.4%), non-specialists (2.7%), and institutions (1.1%). On Bilibili, specialists and individuals each accounted for 47.2% of uploaders, while on TikTok, specialists dominated (75.3%), followed by individuals (22.7%). Non-specialists and institutions were more prevalent on Bilibili (3.4% and 2.2%, respectively) than on TikTok (2.1% and 0%).

**Fig. 3:**
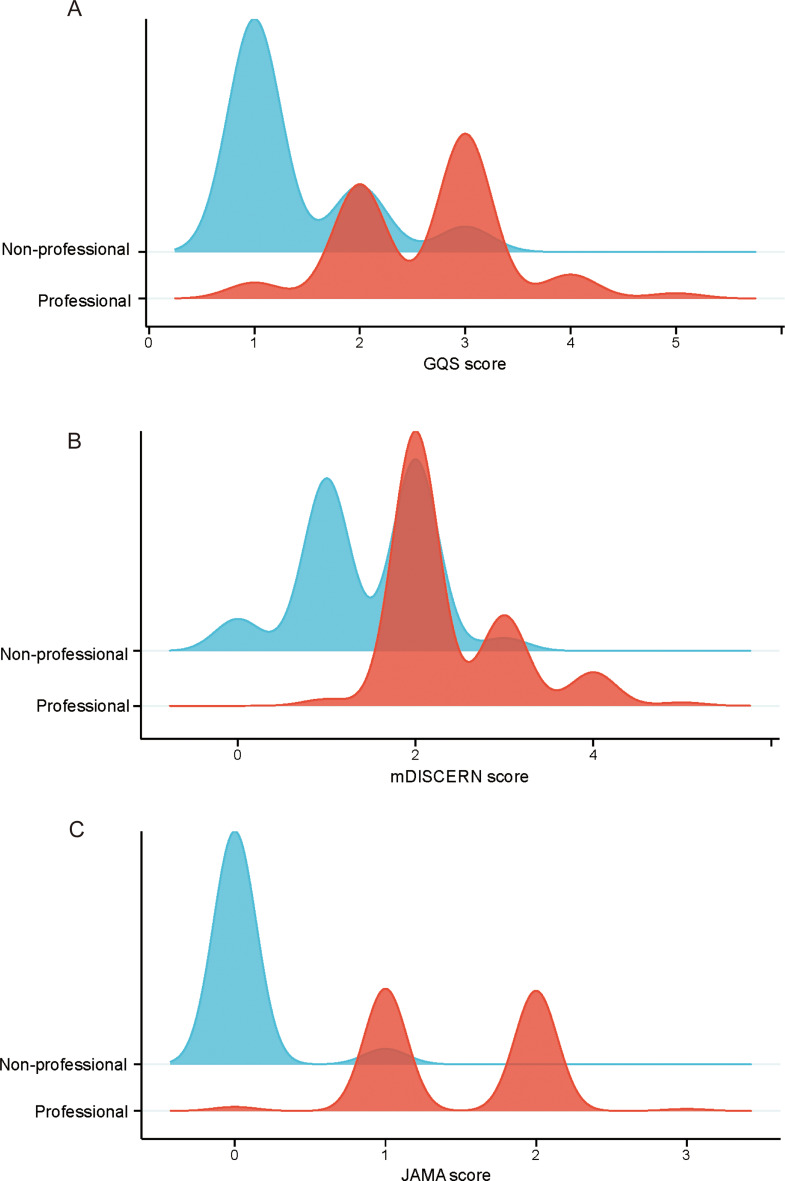
Distribution and comparison of quality and reliability scores across different types of uploader groups. (**A**) Distribution of GQS score. (**B**) Distribution of mDISCERN score. (**C**) Distribution of JAMA score

For further analysis, uploaders were stratified into professionals (specialists, non-specialists, and institutions) and non-professionals (individuals). As shown in Table 2, videos from non-professionals were significantly longer (median: 162 vs. 90 s, *P* = 0.005) but garnered fewer collections (median: 35.5 vs. 90.5, *P* = 0.038) and shares (median: 19.5 vs. 117.5, *P* = 0.033). The distribution and comparison of GQS, mDISCERN and JAMA scores across these uploader groups are presented in Fig. 3. Videos uploaded by professionals performed notably better in quality, with higher median GQS (median: 3 vs. 1, *P* < 0.001), mDISCERN (median: 2 vs. 1.5, *P* < 0.001), and JAMA (median: 2 vs. 1, *P* < 0.001) scores compared to non-professionals.

**Table 2:** Characteristics, quality, and reliability of KD videos by different uploaders on TikTok and Bilibili

Variables	Professionals (*n* = 122)	Non-professionals (*n* = 64)	*P*
Video length(s), M (Q1, Q3)	90(53,164.75)	162(80.25,306)	**0.005**
Likes, M (Q1, Q3)	262.5(32,906.5)	240(8.25,1687.25)	0.43
Collections, M (Q1, Q3)	90.5(18.75,433)	35.5(2.25,342.5)	**0.038**
Comments, M (Q1, Q3)	27.5(2,140.5)	53.5(2.25,424.5)	0.138
Shares, M (Q1, Q3)	117.5(18.25,556.5)	19.5(4,440)	**0.033**
GQS score, M (Q1, Q3)	3 (2,3)	1(1,2)	**< 0.001**
mDISCERN score, M (Q1, Q3)	2(2,3)	1.5(1,2)	**< 0.001**
JAMA score, M (Q1, Q3)	2(2,2)	1(1,1)	**< 0.001**

### Comparison of features across different platforms

**Fig. 4:**
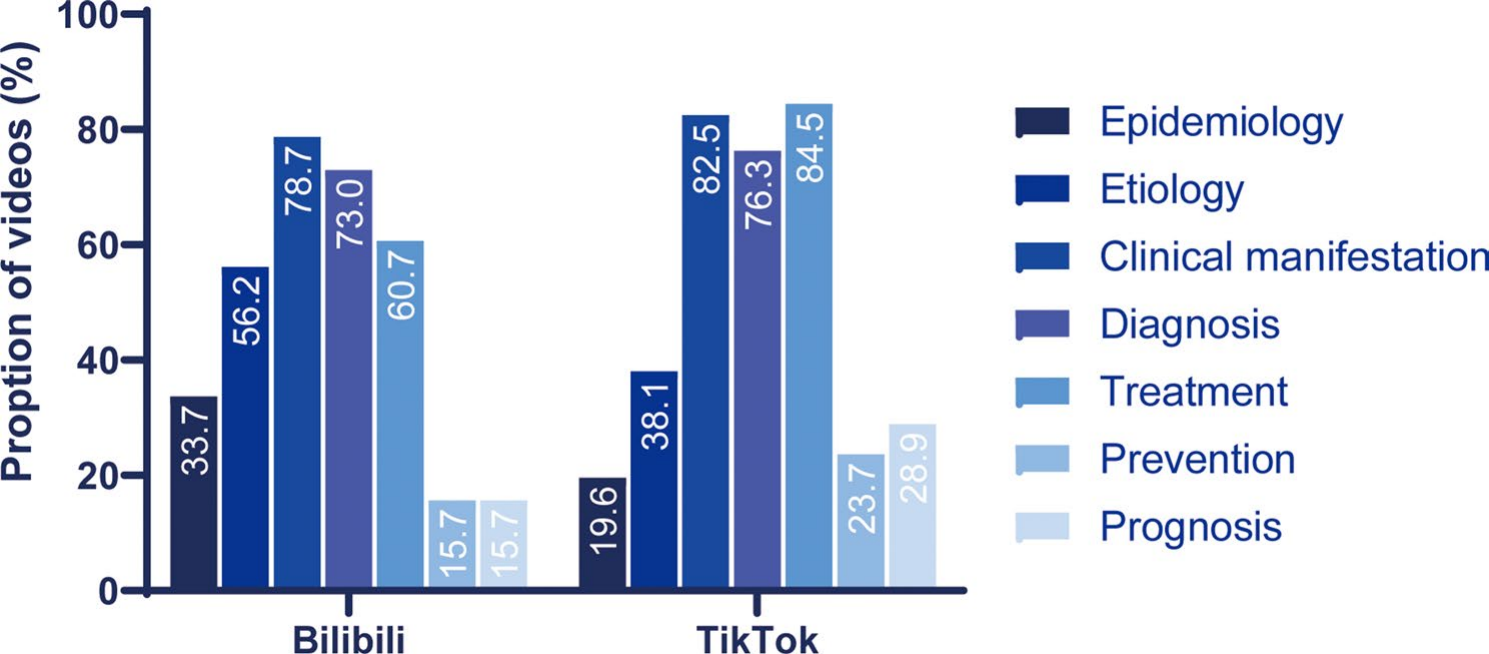
Distribution of contents on Bilibili and TikTok

**Fig. 5:**
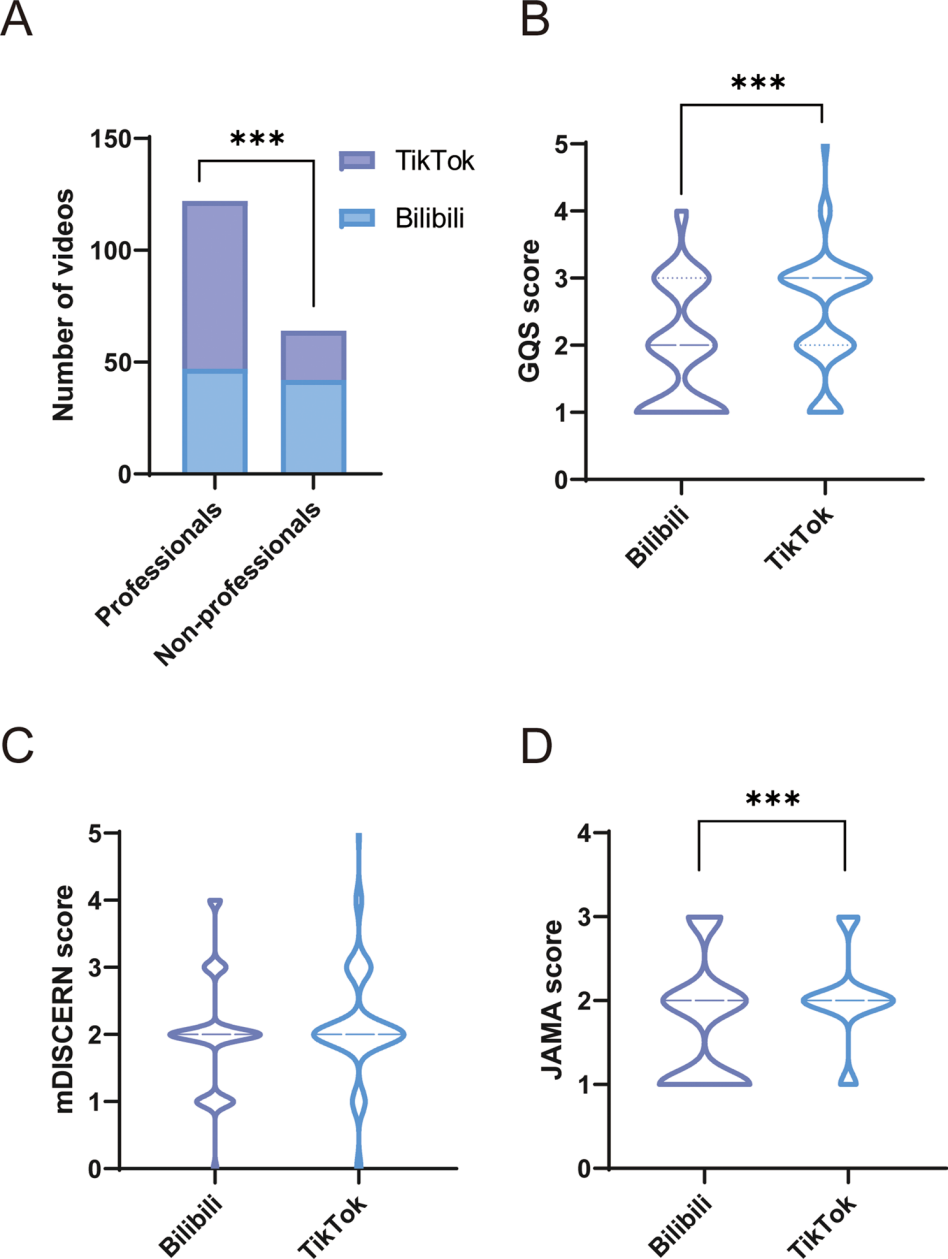
Comparision of video uploaders and quality on Bilibili and TikTok. (**A**) Comparison of uploaders. (**B**) Comparison of GQS score. (**C**) Comparison of mDISCERN score. (**D**) Comparison of JAMA score. ***P < 0.001

Table 3 provides a detailed comparison of video engagement, content, uploader characteristics, and quality between TikTok and Bilibili. Significant differences were observed between the two platforms. Video length was significantly shorter on TikTok than on Bilibili (median: 86 vs. 146 s, *P* < 0.001). In contrast, TikTok videos attracted higher engagement, with more likes (median: 683 vs. 21, *P* < 0.001), collections (median: 243 vs. 12.5, *P* < 0.001), comments (median: 115 vs. 3, *P* < 0.001), and shares (median: 366 vs. 8, *P* < 0.001) than those on Bilibili. As shown in Fig. 4, clinical manifestations, diagnosis and treatment were the most commonly covered topics on both platforms, reported in 78.7%, 73% and 60.7% of Bilibili videos, and 82.5%, 76.3% and 84.5% of TikTok videos, respectively. Treatment (60.7% vs. 84.5%, *P* < 0.001) and prognosis (15.7% vs. 28.9%, *P* = 0.032) were addressed significantly less frequently on Bilibili than on TikTok. In contrast, etiology (56.2% vs. 38.1%, *P* = 0.014) and epidemiology (33.7% vs. 19.6%, *P* = 0.014) were more frequently discussed in Bilibili videos compared to TikTok videos. Additionally, prevention was one of the least covered topic on both platforms, appearing in only 23.7% of TikTok videos and 15.7% of Bilibili videos. In fields of uploaders (Fig. 5A), TikTok had a higher proportion of professional uploaders (77.3% vs. 52.8%, *P* < 0.001). Regarding video quality (Fig. 5B-D), TikTok videos had higher GQS scores (median: 3 vs. 2, *P* < 0.001) and JAMA scores compared to Bilibili videos (median: 2 vs. 2, *P* < 0.001), though mDISCERN scores did not differ significantly between the two platforms (median: 2 vs. 2, *P* = 0.149). Notably, TikTok videos, despite being shorter and more engaging, still exhibited suboptimal quality, suggesting that high engagement does not equate to informational reliability.

**Table 3:** General information, quality, and reliability scores of KD videos on TikTok and Bilibili

Variables	Bilibili (*n* = 89)	TikTok (*n* = 97)	*P*
**General information**			
Video length(s), M (Q1, Q3)	146(68,313)	86(52.5,153.5)	**0.001**
Likes, M (Q1, Q3)	21(4,109.75)	683(264,3190)	**< 0.001**
Collections, M (Q1, Q3)	12.5(1,77.25)	243(66,1107)	**< 0.001**
Comments, M (Q1, Q3)	3(0,20.25)	115(36.5.425)	**< 0.001**
Shares, M (Q1, Q3)	8(1,66)	366(117,1422.5)	**< 0.001**
**Video content**			
Epidemiology	30(33.7%)	19(19.6%)	**0.029**
Etiology	50(56.2%)	37(38.1%)	**0.014**
Clinical manifestation	70(78.7%)	80(82.5%)	0.510
Diagnosis	65(73%)	74(76.3%)	0.610
Treatment	54(60.7%)	82(84.5%)	**< 0.001**
Prevention	14(15.7%)	23(23.7%)	0.173
Prognosis	14(15.7%)	28(28.9%)	**0.032**
**Uploaders**			**< 0.001**
Professional	47(52.8%)	75(77.3%)	
Non-professional	42(47.2%)	22(22.7%)	
GQS score, M (Q1, Q3)	2(1,3)	3(2,3)	**< 0.001**
mDISCERN score, M (Q1, Q3)	2(1,2)	2(2,2)	0.149
JAMA	2(1,2)	2(2,2)	**< 0.001**

### Correlation analysis

**Fig. 6:**
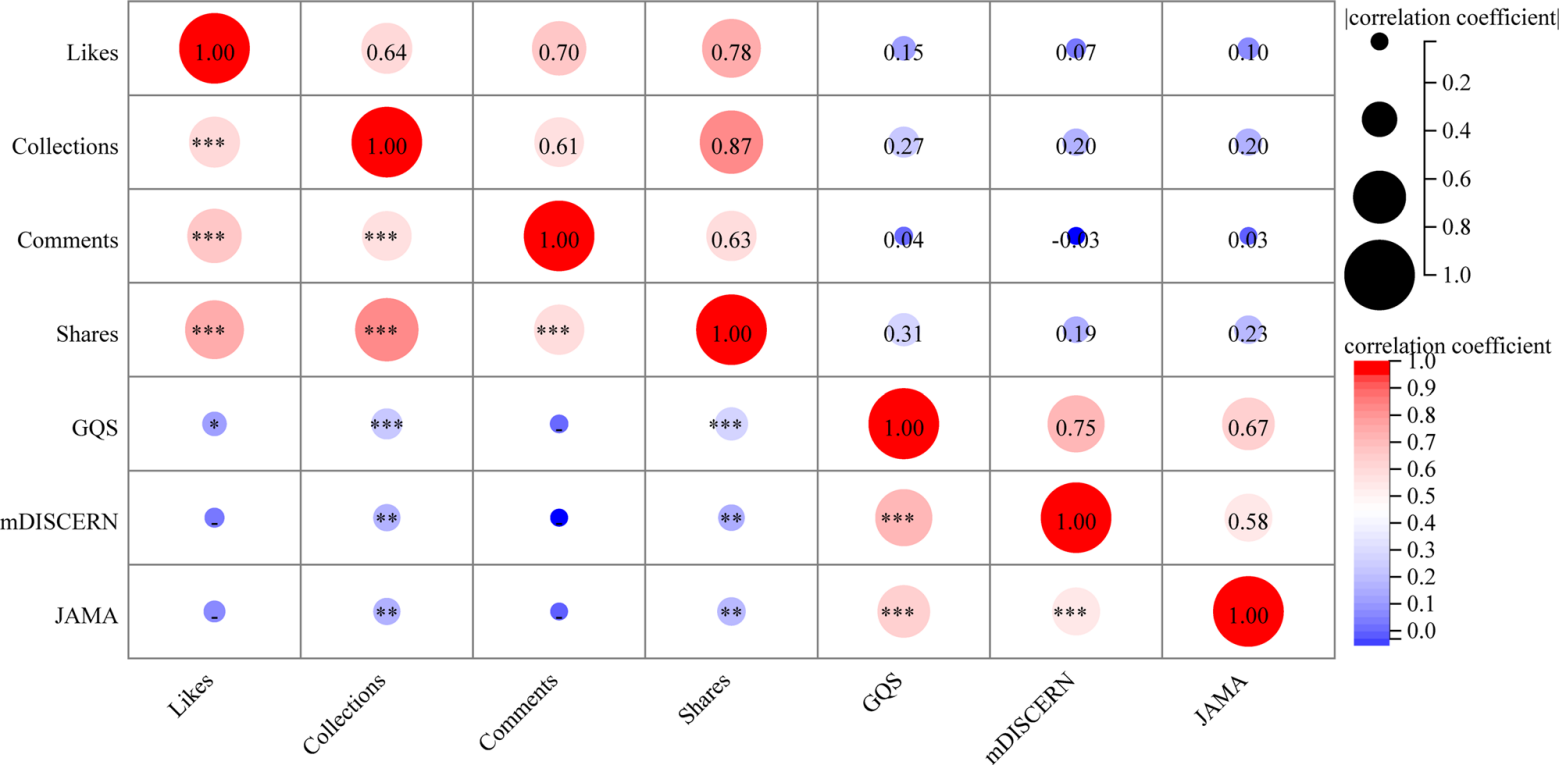
Spearman correlation analysis among different video variables. *P < 0.05, **P < 0.01, ***P < 0.001

Figure 6 presents the correlation analysis between video interaction metrics (such as likes, comments, collections, and shares) and video quality across both TikTok and Bilibili platforms. The results revealed significant positive strong correlations among the interactive metrics. For instance, the correlation coefficients between likes and comments, likes and shares, and likes and collections are 0.7 (*P* < 0.001), 0.78 (*P* < 0.001), and 0.64 (*P* < 0.001), respectively. GQS scores strongly correlated with mDISCERN (ρ = 0.75, *P* < 0.001) and JAMA scores (ρ = 0.67, *P* < 0.001), while mDISCERN and JAMA scores showed a moderate correlation (ρ = 0.58, *P* < 0.001). Conversely, the correlations between interaction metrics and the GQS score or mDISCERN score are extremely weak, bordering on negligible.

## Discussion

This study represents the first systematic evaluation of KD-related content quality on Chinese short-video platforms. Our analysis of 186 videos revealed suboptimal quality, with median GQS, mDISCERN, and JAMA scores of 2, 2, and 2 respectively. These findings indicate a critical gap between widespread platform use for health information and actual content quality available to care-givers seeking guidance on this time-sensitive pediatric condition.

The modest quality scores align with previous research on social media health content [14, 23], yet implications for KD are particularly concerning. Delayed diagnosis beyond 10 days substantially increases coronary artery lesion risk from 5% to over 20% [5]. When caregivers rely on incomplete or misleading information from short videos, they may fail to recognize critical symptoms or underestimate the urgency of seeking medical evaluation, potentially compromising patient outcomes [28].

Our content analysis revealed substantial informational imbalances that limit the educational value of these videos. While clinical manifestations and diagnosis were well-covered, critical topics received inadequate attention: epidemiology was discussed in only 26.3% of videos, prognosis in 22.6%, and prevention in 19.9%. This selective emphasis creates a fragmented understanding of KD. While caregivers may learn to identify symptoms from these videos, the limited coverage of prognosis could create a false sense of security. This, in turn, may lead to delays in necessary follow-up care. Insufficient emphasis on long-term cardiovascular follow-up might cause families to underestimate the critical importance of ongoing echocardiographic monitoring after KD’s acute phase [6, 29]. Furthermore, deficient knowledge regarding epidemiology and preventive measures compromises parents’ capacity for the early identification and intervention of at-risk children [3, 30]. Such content gaps suggest these platforms currently function primarily as symptom awareness tools rather than comprehensive disease education resources. The observed content imbalance likely reflects both platform-specific constraints and algorithmic incentives. Short-video formats inherently favor visually demonstrable content, such as characteristic rashes or conjunctival changes, over abstract epidemiological concepts or long-term prognosis discussions [31].

Transitioning from content characteristics to platform-specific differences, our analysis revealed that TikTok and Bilibili exhibit distinct patterns that reflect their divergent platform architectures, user demographics, and content ecosystems [32]. TikTok videos demonstrated significantly higher GQS and JAMA scores and substantially greater user engagement across all metrics, despite being considerably shorter. This paradoxical relationship between brevity and quality challenges conventional assumptions that comprehensive health education requires extended exposition and suggests that concise, well-structured content may actually enhance information retention and user engagement [33]. The superior performance of TikTok content may be attributable to the platform’s stricter content moderation policies and the higher proportion of professional uploaders, suggesting that platform governance structures directly influence information quality.

The content focus differences between platforms further illuminate distinct user expectations and information-seeking behaviors. Bilibili users encountered more content on etiology and epidemiology, suggesting that this platform’s longer video format and academically oriented user base may facilitate more in-depth explorations of disease mechanisms [23, 34]. Conversely, TikTok’s emphasis on treatment information aligns with its reputation for practical, action-oriented content consumption [34]. These platform-specific content patterns suggest that multi-platform health communication strategies should be tailored to leverage each platform’s strengths rather than applying uniform approaches across all digital channels.

Weak correlations between engagement metrics and quality scores challenge assumptions that high-quality content naturally garners greater visibility. This disconnect suggests that current platform algorithms may not effectively surface the most accurate or comprehensive health information to users seeking guidance on KD. Instead, current algorithms may prioritize emotional appeal over educational value [20, 35, 36], underscoring the need for mechanisms to identify and promote accurate health information through verification systems or algorithmic modifications.

This study has several limitations that warrant acknowledgment and suggest directions for future investigation. First, the cross-sectional design captures content quality at a single time point, precluding assessment of temporal trends in information quality or the impact of emerging platform policies. Longitudinal studies tracking quality evolution could identify whether platform governance interventions or increased professional engagement led to sustained improvements. Second, our analysis focused exclusively on Chinese-language content on Chinese platforms, limiting generalizability to other linguistic and cultural contexts. While TikTok and Bilibili represent dominant platforms in the Chinese digital ecosystem, patterns observed here may not translate to international versions of these platforms or to other video-sharing services. Cross-cultural comparative studies examining KD content across multiple countries and platforms would elucidate whether quality deficiencies represent universal challenges or culturally specific phenomena. Thirdly, this study assessed educational quality and reliability using structured tools but did not verify the factual accuracy of individual video claims. Finally, the cross-sectional design precludes assessment of direct patient outcomes, such as care-seeking delays. Despite the above limitations, our findings have important clinical and policy implications. Pediatricians can serve as‘digital navigators’by guiding families to verified, evidence-based online resources, thereby supporting informed decision-making and improved health outcomes. Healthcare institutions should strategically engage with platforms through verified accounts posting evidence-based content, improving the information ecosystem while building public trust. Platform governance improvements should include professional verification programs, health-specific content guidelines developed with medical organizations, algorithmic modifications prioritizing quality alongside engagement, and mandatory disclosure requirements for creator credentials and information sources.

## Conclusions

This study reveals significant quality and reliability deficiencies in KD-related short videos on TikTok and Bilibili, with particularly concerning gaps in content addressing prognosis, prevention, and disease etiology. Professional uploaders produce higher-quality content than non-professionals, yet substantial room for improvement remains across all uploader categories. The weak correlation between user engagement metrics and content quality highlights a fundamental misalignment between algorithmic amplification and informational value, suggesting that popular content is not necessarily accurate or comprehensive. Platform-specific differences in content characteristics and quality underscore the need for tailored health communication strategies that account for distinct platform cultures and user expectations. Addressing these challenges will require coordinated efforts among healthcare professionals, platform developers, policymakers, and patient advocacy organizations to establish governance frameworks, verification systems, and quality standards that prioritize health information integrity over engagement metrics. We recommend that pediatric healthcare providers and professional organizations play a more proactive role in digital health communication. Collaborations between pediatric societies and platforms such as TikTok and Bilibili should be encouraged to implement ‘verified expert’ labels for urgent pediatric conditions like KD, enhancing the visibility of reliable content. As short-video platforms increasingly serve as primary sources of health information for millions of families worldwide, ensuring the accuracy and completeness of pediatric health content represents both a public health imperative and an opportunity to harness digital media’s potential for improving child health outcomes.

## Data Availability

The original data used in this study were obtained from TikTok and Bilibili and are publicly available on the platforms. The analyzed datasets generated during the study are available from the corresponding author upon reasonable request.

## Abbreviations

CALs: Coronary Artery Lesions
GQS: Global Quality Scale
IVIG: Intravenous Immunoglobulin
KD: Kawasaki Disease
mDISCERN: modified DISCERN tool
